# A Case of Cerebro-Spinal Meningitis, or Cerebro-Spinal Fever—Its Diagnosis, Circumstances of Its Origin, and Treatment

**Published:** 1883-03

**Authors:** John H. Logan

**Affiliations:** Atlanta, Ga., Professor of General and Medical Chemistry in Atlanta Medical College


### A CASE OF CEREBRO-SPINAL MENINGITIS, OR CEREBRO-SPINAL FEVER—ITS DIAGNOSIS, CIRCUMSTANCES OF ITS ORIGIN, AND TREATMENT.

By JOHN H. LOGAN, M.D., ATLANTA, GA.,
Professor of General and Medical Chemistry in Atlanta Medical College.

About five o'clock P.M. of December 16th, 1882, I was called to see Mr. W. F., a railroad employee, residing on West Peters street, aged about twenty-five years; light hair, blue eyes, slender form; pronounced nervous temperament; only a short time married. He had been taken ill a few hours before my visit, after eating a moderate dinner, but had been feeling unwell some time previous. A friend who walked with him after dining to his store heard him complain of being dizzy; disposed to fall backward as he walked, called his attention to this fact once or twice, as he was in the act of steadying himself to avoid falling. Some time after reaching the store, he completely succumbed to the attack, and would have fallen to the floor if his companion had not supported him. He was brought home, unconscious, and convulsed with clonic spasms. I found him speechless and frightfully convulsed, throwing his arms wildly about, struggling to get out of bed, and requiring the strength of several persons to hold him in it. Opisthotonos was obvious in his contorsions, and, whenever in the proper position for it, he would pass his hand over his head and back of the neck, as if pain or some unusual sensation was felt there.

During these muscular exertions he gave utterance to an unearthly, maniacal yell that could be heard several blocks away; pupils dilated and insensible to light, the balls turned somewhat up, imparting to the face a death-like expression; pulse weak and compressible, but in other respects almost normal; respiration 11 to 12 per minute; a profuse perspiration bathed his brow and face, while the hands and feet—especially the former—were cold.

I, unfortunately, was not prepared to take the temperature at any stage of the case. Early in the night he became greatly nauseated, and such agonized retching I never witnessed in any patient before; but after vomiting the partially digested food of the previous meal, this symptom entirely ceased before morning. There was no coma, no delirium, and I made out no hyperæsthesia of the surface and no cutaneous spots. I think that both sight and hearing were lost until late in the progress of the attack. The pulse-beat averaged about 75 or 80 throughout the invasion; was never, except at its beginning, far from its normal conditions. The bowels were costive.

*Diagnosis.*—I was greatly at a loss in ready diagnosis. The father informed me that in his work on the Central Railroad he had been greatly exposed to malaria, and had suffered from chills and fever during the summer previous and part of the late fall. This fact I regarded as important, and hastily decided upon congestion in a malignant intermittent form, despite the season. The cold sweat upon the brow, the anxious face, the terrible nausea, the weak, depressed pulse and spasm, each pointed to malignant, intermittent fever.

*Therapeutics.*—I gave, at once, an enema of aqua ammonia, ʒi, with ℥ij of brandy, and applied, as quickly as they could be prepared, sinapisms to the extremities, hot irons to the feet and bottles filled with hot water to the trunk; injected hypodermically one-quarter of a grain of morphine. The pulse responded at once to this treatment, and the feet became warm, but in other respects there was no favorable change. In half an hour I repeated the enema of brandy and ammonia, and a little later the hypodermic dose of morphine. About nine o'clock I had him stripped and plunged into a bath of hot water, pouring the water over his shoulders and down the spine. Fifteen minutes after, having replaced him in bed and made the surface perfectly dry with a good deal of rough rubbing, a blistering plaster was placed upon the back of the neck, and soon after it was extended some distance down the spine. But before this, the true diagnosis of the case had dawned upon me. There seemed to be some amendment in the symptoms, in longer intervals occurring between the spasms, but these, with the yells, continued.

I now ordered a solution of potassium bromide and chloral hydrate, sixteen grains of the first and twelve of the latter, every hour. By two o'clock A.M., the spells of quiet rest and the intervals between the convulsive attacks were markedly increased, some thirty minutes intervening. A little before two o'clock, ordering the solution to be continued, I left to seek some rest at my own home.

Called, at about nine o'clock the following morning, the 17th—the patient nearly in the same condition, apparently, in which I had left him, convulsions still persisting after an interval of repose.

I now replaced the bromide and chloral with inhalations of chloroform and found that it answered promptly every expectation; a single sniff of the anæsthetic sending the patient into a profound and apparently refreshing sleep, and again as often as the symptoms called for its use.

The blistering plaster on the back of the neck had drawn well, and the one along the interscapular spine had reddened the surface, and soon gave a good vesication. Half after nine met Dr. J. F. Alexander in consultation; Dr. Whitfield, of Burke county, father-in-law of the patient, had also arrived that morning and heartily sanctioned the treatment then proposed—gave the patient, at once, ten grains saccharated calomel, to be followed every two or three hours with five grains of quinine and one grain of pulverized opium.

At four o'clock in the afternoon, visited him again, alone—ordered a clyster of soap-suds to relieve the bowels—it was obvious that a great amendment had taken place in the patient—consciousness partially restored, no more convulsions—rose from the bed at his own silent suggestion and relieved his bladder in the right vessel—looked intelligently around and spoke several connected words.

In half an hour the clyster acted in a moderately large, well moulded, natural dejection. At night the calomel acted, and he had taken, I think, two papers of the quinine and opium—the opium was now withdrawn and the quinine continued. I saw the patient the last time the next morning the 19th, he was sitting up in bed, his mind fully restored, conversing with his wife. In a week he had returned to his work on the railroad.

